# Exploiting hydrogenases for biocatalytic hydrogenations

**DOI:** 10.1039/d4cc04525d

**Published:** 2024-10-29

**Authors:** Daria Sokolova, Kylie A. Vincent

**Affiliations:** a Department of Chemistry, University of Oxford, Inorganic Chemistry Laboratory South Parks Road Oxford OX1 3QR UK kylie.vincent@chem.ox.ac.uk

## Abstract

The ability of hydrogenase enzymes to activate H_2_ with excellent selectivity leads to many interesting possibilities for biotechnology driven by H_2_ as a clean reductant. Here, we review examples where hydrogenase enzymes have been used to drive native and non-native hydrogenation reactions in solution or as part of a redox cascade on a conductive support, with a focus on the developments we have contributed to this field. In all of the examples discussed, hydrogenation reactions are enabled by coupled redox reactions: the oxidation of H_2_ at a hydrogenase active site, linked electronically (*via* relay clusters in the enzyme and/or *via* conductive support) to the site of a reduction reaction, and we note how this parallels developments in site-separated reactivity in heterogeneous catalysis. We discuss the productivities achieved with biocatalytic hydrogenations, the scope for application of these approaches in industrial biotechnology, possibilities for scaling the production of hydrogenases, and future opportunities. Our focus is on NiFe hydrogenases, but we discuss briefly how FeFe hydrogenases might contribute to this field.

## Abstract 2

The ability of hydrogenase enzymes to activate H_2_ with excellent selectivity leads to many interesting possibilities for biotechnology driven by H_2_ as a clean reductant.

## Graphical abstract

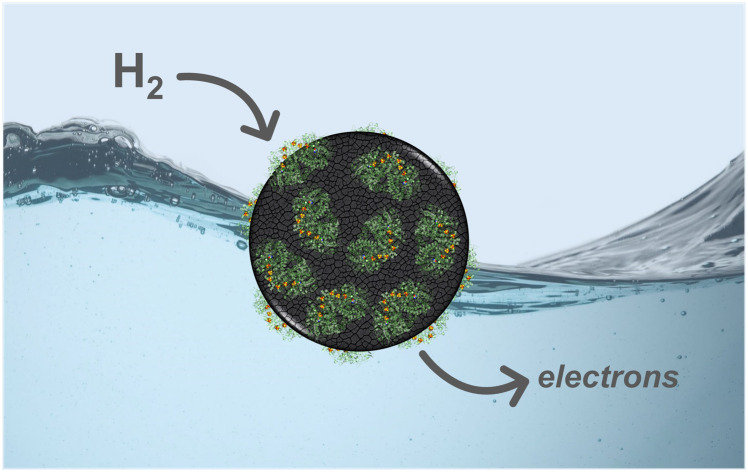

## Introduction

In the 1930s, Stevenson and Stickland^1^ noted the ability of hydrogenase-containing microbes to pass electrons from H_2_ to a variety of acceptors: ‘By means of this enzyme, hydrogen reduces molecular oxygen, methylene blue, nitrate and fumarate’. We now know that the biological roles and forms of hydrogenases are numerous. The basic protein subunits housing the NiFe or FeFe active site of hydrogenases are found in simple, soluble proteins through to multi-subunit complexes, linking oxidation of H_2_ to the biological reduction of nitrate, O_2_, the nicotinamide cofactors NAD(P)^+^, the deazaflavin coenzyme F_420_,^2^ and many other acceptors. Here, we explore how the ability of hydrogenase to pass on electrons from H_2_ has inspired a suite of new biotechnology approaches which couple the oxidation of H_2_ to various reductions. These extend beyond native enzyme activities by coupling hydrogenase from one organism to a reductase from another, linking enzymes *via* electronically conductive supports, and demonstrating new-to-nature activity for hydrogenases. These developments are timely as the fine chemical industry seeks greener processes which minimise chemical waste, and hydrogenation is seen as a clean alternative to stoichiometric reductions.^3^ This has led to interest in H_2_-driven biocatalysis.^4^ NiFe hydrogenases have proved the most amenable to application in biotechnology because of their greater stability in air – in some cases, they can sustain H_2_ oxidation in the presence of O_2_ – and longer lifetimes. We, therefore, focus largely on the NiFe enzymes, although we briefly discuss opportunities for hydrogenations using FeFe hydrogenases.

Development of hydrogenation catalysts which couple biological oxidation of H_2_ to a site-separated reduction process is mirrored in recent mechanistic advances in heterogeneous catalysis where certain hydrogenation reactions have been shown to occur *via* H_2_ oxidation at Pd with reduction of a substrate at a separate metal site or at conductive support, and we note where there are parallels.

Biotechnology for chemical manufacturing has developed rapidly, and is of particular interest in the pharmaceuticals and agrochemicals sectors where stereoselective biocatalysis offers significant advantages for generating chiral products.^5,6^ However, biocatalytic processes are being re-evaluated in light of the growing drive for more sustainable manufacturing practices.^4,7^ Many of the oxidoreductases employed in asymmetric synthesis are dependent on nicotinamide cofactors, NADH or NADPH, as a source of reducing equivalents. Since the delivery of reducing equivalents from these cofactors occurs *via* direct hydride transfer in an enzyme active site pocket, it has proved difficult to substitute more accessible electron donors or acceptors. The efficient recycling of NAD(P)H cofactor using glucose dehydrogenase (GDH) with glucose as a sacrificial hydride donor has enabled the expansion of biotechnology for chemical manufacturing. However, dependence on the (super)-stoichiometric levels of the C_6_ sugar molecule, glucose, as a hydride donor is becoming increasingly troubling due to the waste accumulated, and more atom-economical reductants are desirable.^4^ The cost of glucose is also a factor and has contributed to biocatalysis being confined largely to the manufacture of high-value chemicals. It has proved difficult to find non-biological methods for recycling the oxidised or reduced cofactors, NAD(P)^+^ or NAD(P)H. There have been developments in transfer hydrogenation of NAD(P)^+^ from formate or H_2_ using Ir or Rh complexes,^8,9^ or Pt nanoparticles^10^ and in electrochemical regeneration,^11^ but as yet these have been limited in application, often forming some incorrect cofactor during each turnover cycle, and remaining dependent on precious metals. The field of chemo-catalysed NAD(P)H recycling remains active and may contribute to industrial approaches in future. The high cost of the cofactors means that even 0.1–1% incorrect NAD(P)H (with the hydride on the wrong position or in dimeric form) from each reaction cycle makes it impossible to achieve the >1000–10 000 cofactor turnovers which are needed to make a biocatalytic process economically viable. For this reason, H_2_-driven biocatalytic NAD(P)H recycling is attracting attention and we show a number of ways in which hydrogenases may contribute in this arena. We also show that the scope for hydrogenases in chemical synthesis goes far beyond NAD(P)H recycling. We review recent work which opens up possibilities for biocatalytic hydrogenations *via* flavin recycling and by direct reductions at a conductive support.

Biocatalytic hydrogenation could ‘slot in’ readily to existing chemical manufacturing, where heterogeneous or homogeneous hydrogenations are already firmly embedded and account for around 10–20% of all industrial chemical steps.^12^ Obviously, increasing scope for H_2_-driven chemical manufacturing demands new ways of scaling the clean production of H_2_*via* electrolysis of water powered by solar and wind energy, and such technologies are also immature.^13^ However, in a future sustainable energy economy, electrically-driven production of H_2_ for clean chemical manufacturing could become an important way to utilise and store electricity during peak periods of production. New avenues in H_2_-driven biotechnology which we describe here will help to ensure that the industrial biotechnology sector is ready to exploit an emerging renewable energy economy in which H_2_ is a significant energy vector.

### Mechanism, inhibition, and selectivity of NiFe hydrogenases

**Fig. 1:**
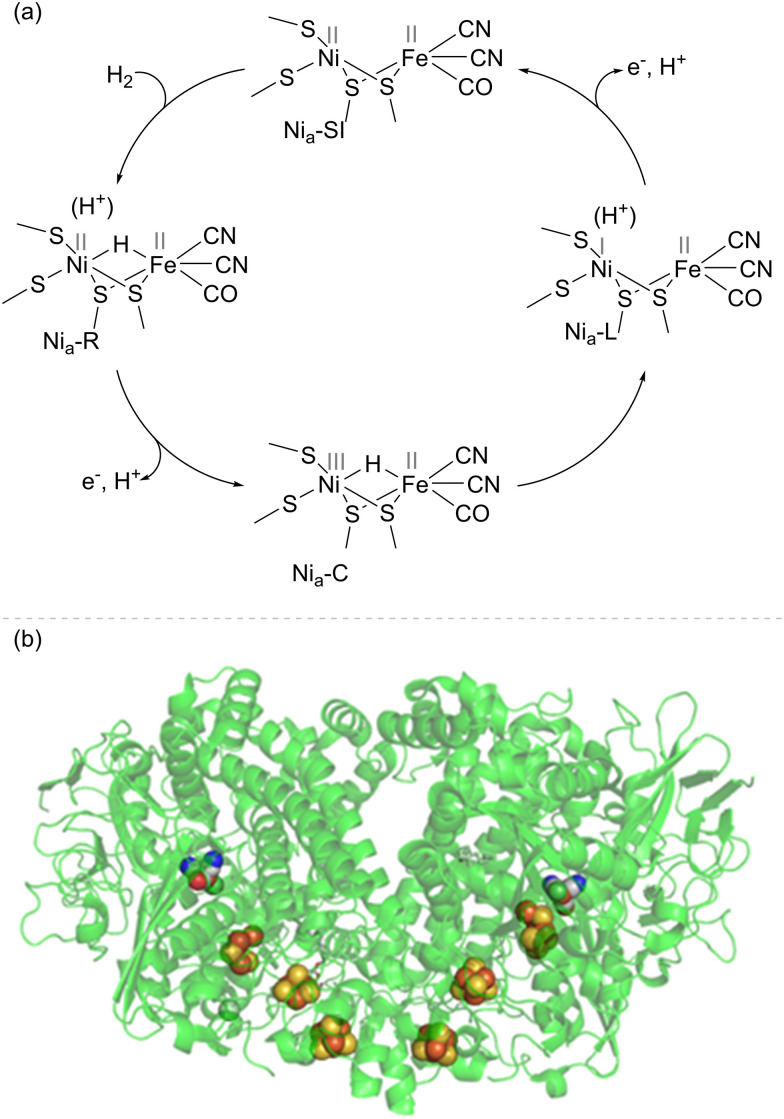
(a) Basic catalytic cycle for NiFe hydrogenases. This is viewed in the direction of H_2_ oxidation, although the steps are likely to be reversible, and many NiFe hydrogenases are able to evolve H_2_ at appropriate potentials. (b) Structure of NiFe hydrogenase I (Hyd-1) from *E. coli*, showing the [NiFe]-active site, and [FeS] electron relay clusters as spheres. *E. coli* Hyd-1 exists as a dimer, where each half comprises a small and large subunit with the active site and [FeS] electron-relay chain (shown as spheres in elemental colours).

The most well-studied NiFe hydrogenases have a large subunit housing the [NiFe]-active site and a small subunit housing an electron-relay chain of iron–sulfur clusters. It is likely that the main mechanistic features of H_2_ activation (Fig. 1) are common across the NiFe hydrogenases, although there may be differences in proton transfer pathways and the extent to which proton and electron transfers during catalysis are concerted.^14^ Heterolytic cleavage of H_2_ at the Ni(ii)Fe(ii) level of the active site (labelled the Ni_a_-SI state) gives a hydride-bridged Ni(ii)(H^−^)Fe(ii) species (Ni_a_-R) which is presumably protonated near the active site. Proton and electron transfer away from the active site yields a Ni(iii)(H^−^)Fe(ii) intermediate (Ni_a_-C), which tautomerises to a Ni(i)Fe(ii) active site with nearby protonation (Ni_a_-L). A further cycle of proton and electron transfer re-generates the Ni(ii)Fe(ii) Ni_a_-SI starting state. Each cycle of catalysis sends two electrons, one at a time, to the electron relay chain of iron–sulfur clusters.^14^ Depending on the cellular role of the hydrogenase, electrons may pass to a membrane-bound cytochrome, where they are ultimately transferred *via* the quinone/quinol pool to a reductase to drive the reduction of O_2_ or nitrate, for example. More complex NiFe hydrogenases have built-in reductase modules, such as a flavin catalytic site for NAD(P)^+^ or F_420_ reduction. The NiFe hydrogenase 1 (Hyd-1) from *E. coli* (used extensively in the biotechnology examples discussed in this Feature Article) exists as a dimer, such that the two electron transfer chains (from two [NiFe]-active sites) meet at the same surface of the protein (Fig. 1(b)), with electrons transferred *in vivo* to a cytochrome subunit.

One of the aspects of hydrogenase catalysis which has intrigued chemists is the high selectivity of these enzymes for H_2_ over small molecules that are typical poisons of precious metal catalysts. For example, the O_2_-tolerant NiFe enzymes are almost completely insensitive to poisoning by CO during H_2_ oxidation, and NiFe hydrogenases have been shown to recover easily from reaction with H_2_S.^15^ The most effective metallic hydrogenation catalysts (usually based on Pt-group metals) are highly reactive to many unsaturated bonds and hence tend to give poor selectivity in hydrogenation of molecules with multiple unsaturated bonds.^16–20^ The ability of hydrogenases to activate H_2_ selectively, without indiscriminately hydrogenating unsaturated bonds, opens up new mechanistic possibilities in hydrogenation catalysis where the enzymes can be used purely to provide a supply of electrons from H_2_, and these can be used at a separate site for a reduction process.

## Applications of hydrogenases

### Applications of soluble NAD(P)^+^-linked hydrogenases for NAD(P)H recycling

**Fig. 2:**
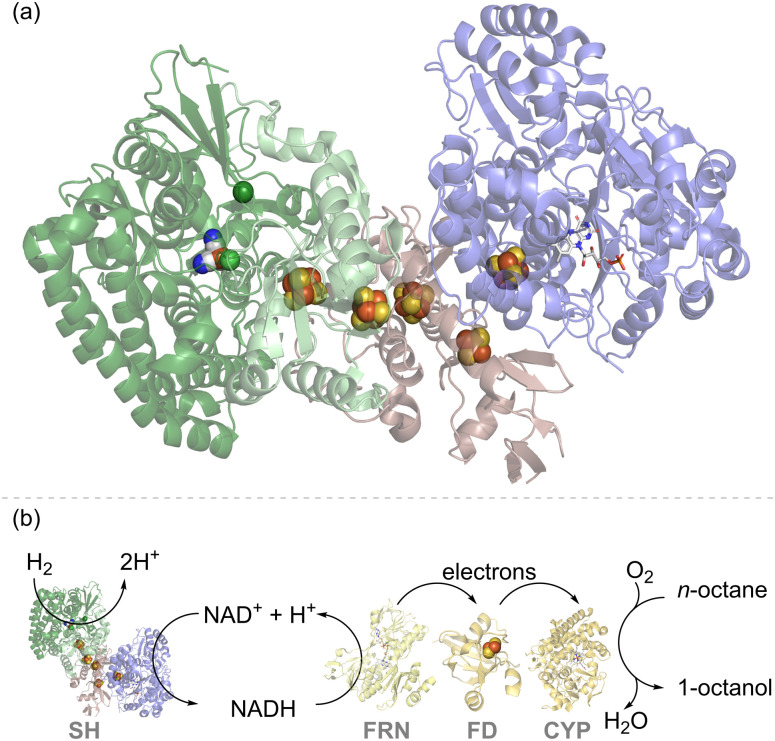
(a) Structure of the soluble hydrogenase (SH) from *Hydrogenophilus thermoluteolus* showing the hydrogenase moiety in green, with the NiFe(CO)(CN)_2_ catalytic site shown as spheres in elemental colours, and the NAD^+^/NADH cycling moiety in blue, with the flavin catalytic site shown in stick form. Iron–sulfur electron relay clusters are shown in spheres in elemental colours.^21^ (b) An engineered strain of *Pseudomonas putida* expressed SH and cytochrome P450 monooxygenase (CYP) together with the NADH-ferredoxin reductase (FRN) and ferredoxin (FD) needed for electron transfer. This enables enhanced activity for octane to octanol in the presence of H_2_, attributed to H_2_-driven NAD^+^ reduction by the SH, as described in the ref. 22.

Fig. 2(a) shows the structure of the ‘soluble hydrogenase’ (SH) from *Hydrogenophilus* (*H.*) *thermoluteolus* which natively links the redox half reactions of H_2_ oxidation (eqn (1)) and NAD^+^ reduction (eqn (2)), to give the overall reaction shown in eqn (3), or the reverse reaction in which NADH oxidation is coupled to H_2_ evolution.^21^

1 H_2_ → 2H^+^ + 2e^−^

2 NAD^+^ + 2e^−^ + H^+^ → NADH

3 H_2_ + NAD^+^ → NADH + H^+^

Physiologically, this enzyme allows organisms such as *Cupriavidus necator* (*C. necator*, formerly known as *Ralstonia eutropha*) or *H. thermoluteolus* to store reducing equivalents from H_2_ in chemical form, as NADH. The hydrogenase moiety (green) contains a typical [NiFe]-catalytic site (Fig. 1(a)), linked *via* a chain of iron–sulfur clusters to a flavin active site for NAD^+^/NADH cycling in the NAD^+^-reductase moiety (blue). Both catalytic sites work reversibly, *i.e.* operating at the thermodynamic potential for the H^+^/H_2_ couple (−0.413 V at pH 7.0 and 1 bar H_2_) and NAD^+^/NADH couple (−0.320 V at pH 7.0 and a 1 : 1 ratio of NAD^+^ : NADH), respectively. The close spacing of these redox couples in potential (voltage) means that both directions of reaction (H_2_ to NADH or NADH to H_2_) are thermodynamically favourable upon a slight variation in conditions. For example, for a solution in equilibrium with a much lower level of H_2_ (0.1%) the potential of the H^+^/H_2_ couple at pH 7.0 shifts in a positive direction to −0.325 V. At a 10-fold excess of NADH to NAD^+^, the NAD^+^/NADH couple shifts in a negative direction to −0.350 V. Under these modified conditions, oxidation of NADH by protons is now thermodynamically favoured (the reverse of eqn (3)).

In one of the earlier attempts to develop H_2_-driven biotechnology with NiFe hydrogenase, the NADP^+^-reducing SH from the hyperthermophilic organism, *Pyrococcus furiosus*,^23^ was explored as an NADPH recycling system, but challenges in stability and expression have hindered further applications of this enzyme.^24^ The NAD^+^-linked SH from *C. necator* is O_2_-stable and has been demonstrated for H_2_-driven NADH recycling both in whole-cell biocatalysis and *in vitro*.^22,25,26^ For example, cells of *Pseudomonas putida* were modified for heterologous expression of an SH and were shown to give a higher rate of *in vivo* cytochrome P450 monooxygenase (CYP)-catalysed octane oxidation to octanol under H_2_, which was attributed to SH-catalysed recycling of NADH (Fig. 2(b)).^22^ Although the cytochrome P450 reaction on octane is an oxidation, it relies upon NADH supply for partial reduction of O_2_ and hence is supported by H_2_-driven NADH recycling.

Other cytochrome P450 monooxygenases are specific for the phosphorylated derivative, NADPH. Site-directed mutagenesis of the SH from *C. necator* enabled a switch in selectivity for NADP^+^ compared to NAD^+^, giving a double variant E341A/S342R with Michaelis Menten constant (*K*_M_) for NADP^+^ of 0.6 mM, very close to that of the wild-type enzyme for NAD^+^.^25^ This system was exploited in purified form for NADPH supply to an isolated NADPH-dependent imine reductase and cytochrome P450 (BM3-type) monooxygenase.^25^ Although these examples are conceptually important, productivity was not sufficiently high to encourage rapid up-scaling and further development.

An attractive concept for mild oxidations is to use protons as a clean oxidant, with the capture of H_2_ as a bonus by-product. This has been demonstrated by Al-Shameri *et al.* with reverse operation of the *C. necator* soluble hydrogenase for an enzyme cascade for D-xylose conversion to α-ketoglutarate requiring two equivalents of the oxidised cofactor, NAD^+^.^27^ Gaseous H_2_ could be detected in the stream flushed out of the reaction vessel. With process improvements to enhance gas removal and capture, this may become an interesting strategy for NAD(P)^+^ dependent oxidative catalysis, with bonus production of H_2_.

### Combining hydrogenase with reductase on a carbon support

**Fig. 3:**
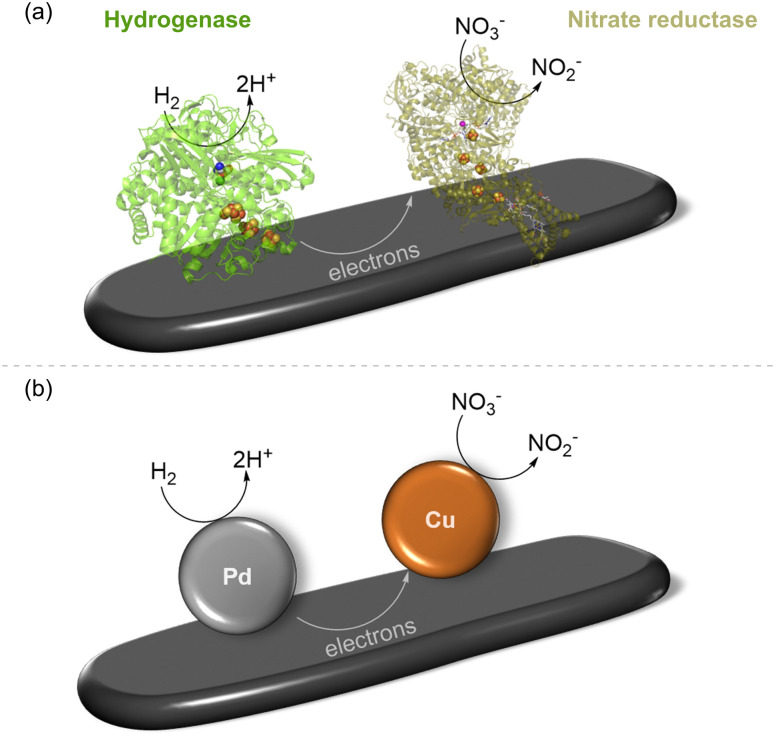
Reduction of nitrate to nitrite by H_2_ using two-site H_2_ oxidation/nitrate reduction catalysis. (a) Biocatalyst system involving NiFe hydrogenase co-immobilised with nitrate reductase on graphite platelets, as reported in ref. 28 (b) schematic representation of a heterogeneous supported metal alloy in which H_2_ oxidation is shown to take place on Pd sites and nitrate reduction to occur on Cu sites, as studied in ref. 29.

Recognising the opportunities offered by the direct flow of electrons between hydrogenase and a carbon surface, in 2007, Armstrong and coworkers coupled hydrogenase with nitrate or fumarate reductase on platelets of graphite and showed that electrons passing through the electronically conductive graphite could drive a reduction.^28^ This is shown schematically in Fig. 3(a) with a single pair of enzymes on the support, although, of course, many molecules of each enzyme will be adsorbed, likely in a random distribution. The NiFe hydrogenase (here, from the purple sulfur bacterium, *Allochromatium vinosum*) oxidises H_2_(eqn (1)) and provides electrons into the conduction band of the graphite, where they can be taken up by nitrate reductase (from *E. coli*) for reduction of nitrate to nitrite (eqn (4)), to give the overall reaction shown in eqn (5). This was confirmed by a colourimetric assay for nitrite.

4 NO_3_^−^ +2H^+^ + 2e^−^ → NO_2_^−^ + H_2_O

5 H_2_ + NO_3_^−^ → NO_2_^−^ + H_2_O

Interestingly, the same overall reaction has been demonstrated recently by Surendranath and coworkers at a heterogeneous PdCu alloy catalyst (shown schematically in Fig. 3(b)) where Pd sites are responsible for an H_2_ oxidation half-reaction (eqn (1)) and Cu sites carry out the nitrate reduction half-reaction (eqn (4)).^29^

### Heterogeneous biocatalytic NADH recycling

**Fig. 4:**
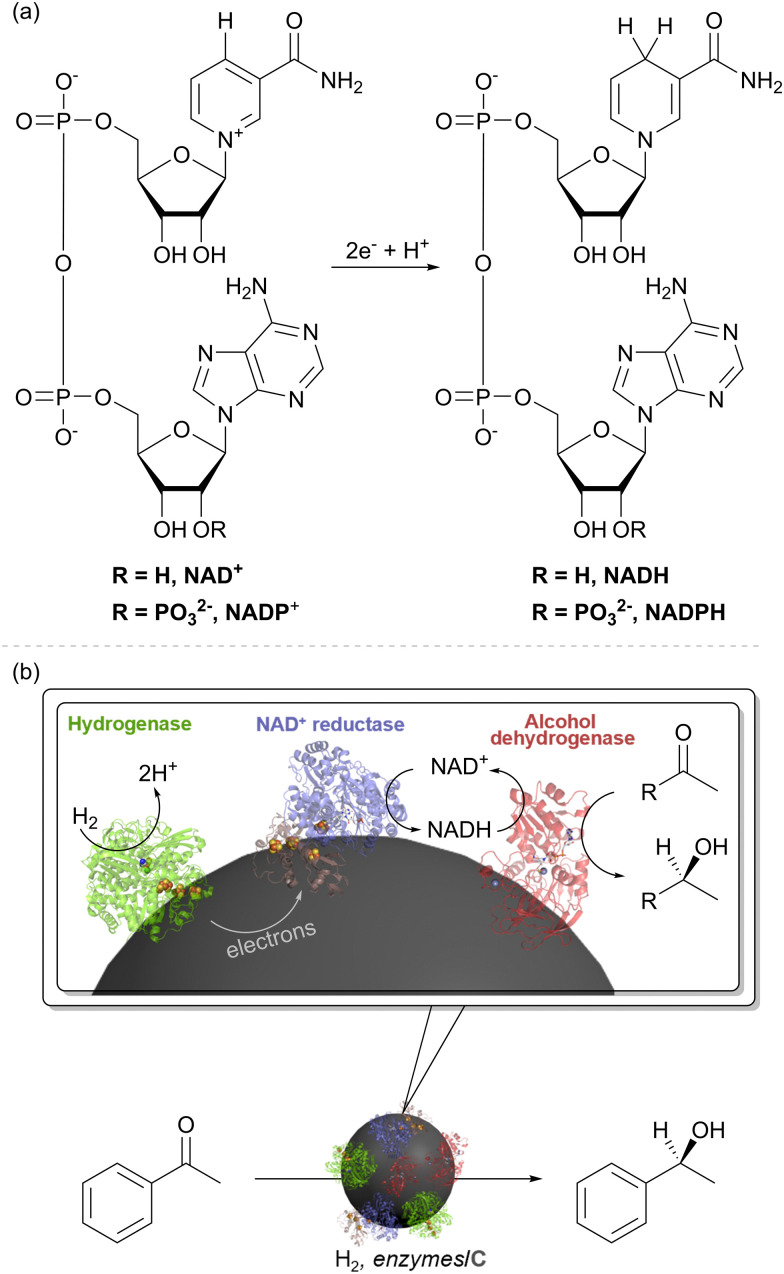
(a) Structures of the nicotinamide cofactors, NAD(P)^+^ and NAD(P)H. (b) An enzyme cascade on carbon particles for biocatalytic hydrogenations. The hydrogenase catalyses oxidation of H_2_ and relays electrons into the conductive carbon support. The NAD^+^ reductase takes up these electrons for NAD^+^ reduction to NADH. An NADH-dependent enzyme such as alcohol dehydrogenase (keto reductase) then takes up NADH for stereoselective reduction of an unsaturated bond, such as reduction of a ketone to an alcohol.

We took up the concept of coupling H_2_ oxidation and a site-separated reduction to develop a heterogeneous catalyst system for H_2_-driven NADH recycling.^30^ The oxidised and reduced nicotinamide cofactors are shown in Fig. 4(a). In this recycling system for NADH, the hydrogenase oxidises H_2_ and provides electrons to the NAD^+^ reductase moiety (*via* the carbon support) for reduction of NAD^+^ to NADH (Fig. 4(b)). The NADH is then available to an NADH-dependent reductase (or ‘dehydrogenase’ working in reverse), for reduction of an unsaturated bond. As an example, this is shown in Fig. 4(b) with an alcohol dehydrogenase (also known as a keto reductase) for the synthesis of a chiral alcohol.^31^ A very wide range of commercial alcohol dehydrogenases are available and have been engineered to act on specific substrates or to exhibit broad substrate scope.^32–34^ Selectivity for generating (*S*)- or (*R*)- alcohols is generally high within a specific enzyme, but commercial alcohol dehydrogenases have been engineered for opposing stereochemical outcomes, and hence it is often possible to generate either enantiomer of a product with high stereoselectivity.^35,36^

Although the overall cofactor recycling reaction is the same as that catalysed by the native SH enzyme discussed above, the modularity of the approach shown in Fig. 4(b) makes it possible to pick the enzyme components for specific reaction requirements. This concept has proved highly successful with the NiFe hydrogenases from the common bacterium, *E. coli*, together with the NAD^+^ reductase moiety of an SH for H_2_-driven NADH recycling (blue/brown subunits of Fig. 2(a)) or the whole SH.^37^ Synthesis of the pharmaceutical building block (3*R*)-quinuclidinol was intensified in continuous flow over a catalyst comprising hydrogenase and NAD^+^ reductase on activated charcoal using an alcohol dehydrogenase from *Agrobacterium tumefaciens* for example.^38^

In an experiment in which immobilised hydrogenase and NAD^+^ reductase were physically separated by a carbon paper layer, the redox state of the hydrogenase active site was confirmed to respond to changes in NAD^+^/NADH ratio (which alter electron flow from the NAD^+^ reductase), showing that the two enzymes were electronically ‘wired’ *via* the conductive support.^39^ Using the bidirectional NiFe hydrogenase, Hyd-2 from *E. coli*, the system has also been run in reverse for NAD^+^ recycling (making H_2_ as a bonus by-product).^39^

The heterogeneous nature of the biocatalytic system for H_2_-driven NADH recycling has enabled translation into continuous flow by loading catalyst particles into a packed bed reactor. Reactions run in the H-cube flow reactor with H_2_ produced by electrolysis of water showcase applicability in an industrial-standard, scalable flow reactor, as well as scope for running on H_2_ produced renewably from water.^37^

### Applications of H_2_-driven NADH recycling in selective deuteration reactions

**Fig. 5:**
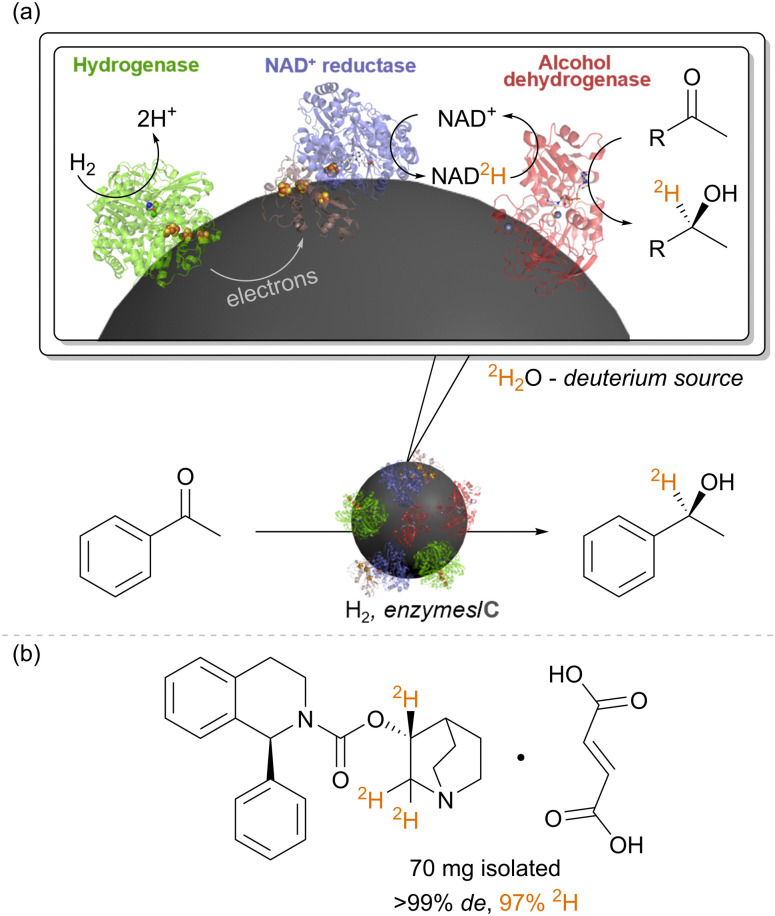
(a) The heterogeneous biocatalytic NADH recycling system can be re-purposed for selective insertion of ^2^H labels into organic chemicals adjacent to the bond which is reduced. (b) This was showcased for selective deuteration of the pharmaceutical solifenacin fumarate, as described in ref. 40.

We have also repurposed the heterogeneous catalyst system of hydrogenase (Hyd-1) and NAD^+^-reductase for biocatalytic insertion of the hydrogen isotope, deuterium, ^2^H, into selective positions on molecules (Fig. 5).^40^ In catalytic conversion of NAD^+^ to NADH by the NAD^+^ reductase, the hydride on NADH derives from a proton in solution, and therefore, running reactions in heavy water, ^2^H_2_O, results in formation of the deuterated cofactor, NAD^2^H (the ^2^H is highlighted in orange in Fig. 5). When supplied to a reductase, such as alcohol dehydrogenase, NAD^2^H results in transfer of the deuteride onto the product, adjacent to the bond which has been reduced. When used with an alcohol dehydrogenase, this results in alpha-deuterated alcohols, for example. (Of course, the –OH group is also deuterated (–O^2^H) but here the deuteron is exchangeable in water.) Again, the stereochemical outcome of the reaction is controlled by the alcohol dehydrogenase. Since the H_2_ oxidation half-reaction is site-separated from the NAD^+^ reduction, the overall reaction proceeds in ^2^H_2_O under unlabelled H_2_ gas, with only slight dilution of the deuterium content of the solvent by protons released by the hydrogenase. We showcased the synthesis of a range of deuterated molecules using this approach, including site-selective deuteration of the pharmaceutical, solifenacin, a muscarinic M3 receptor antagonist. Deuterated pharmaceuticals have in some cases been shown to have better metabolic stability, allowing lower dosage and minimised side effects, and this simple biocatalytic strategy for precision introduction of deuterium atoms from ^2^H_2_O as a cheap ^2^H source is likely to be useful in specific cases of pharmaceutical synthesis, particularly where there are already biocatalytic steps in the synthesis. We have also made use of the intact soluble hydrogenase from *C. necator*, *in vitro*, for recycling the deuterated cofactor NAD^2^H during the synthesis of isotopically-labelled amino acids to use in the expression of labelled proteins for NMR structural study.^41^

### Application of hydrogenases in flavin recycling to support catalysis by ene reductases and nitro reductases

**Fig. 6:**
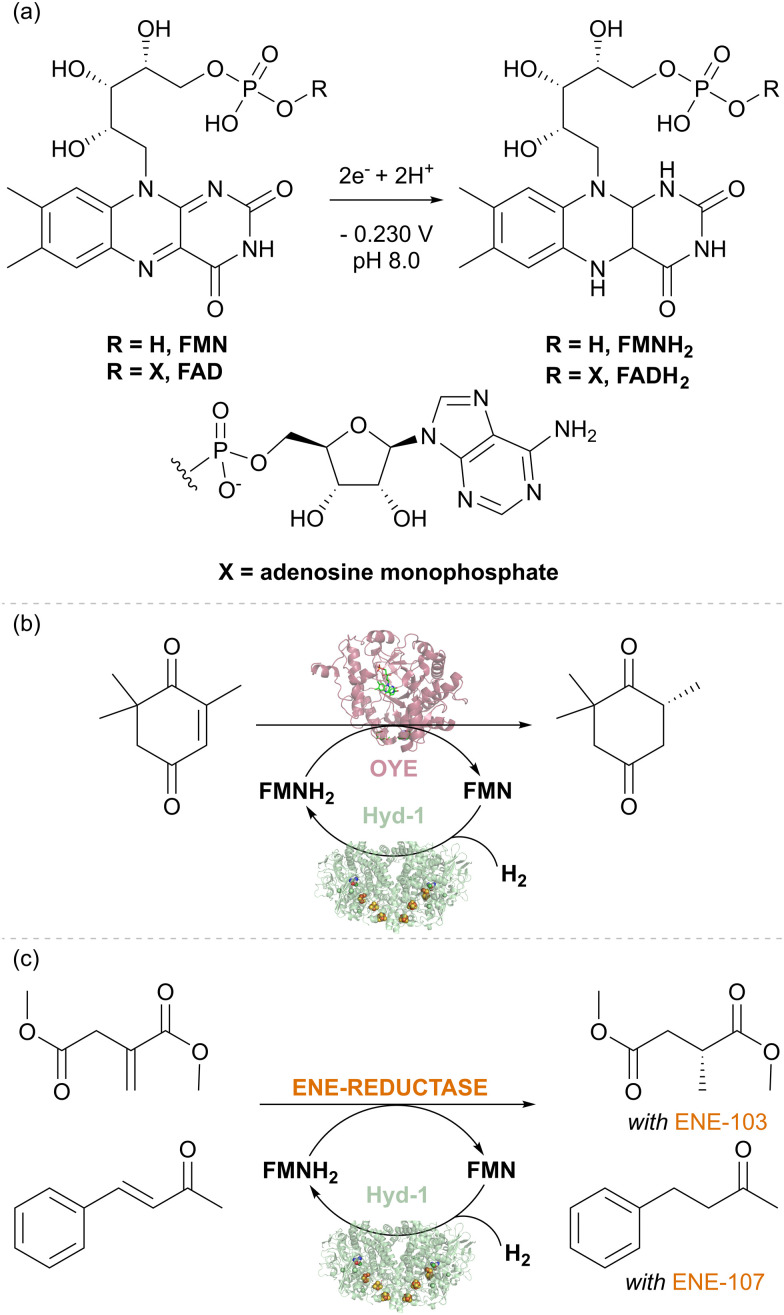
(a) Reduction of flavin cofactors. (b) Stereoselective biocatalytic H_2_-driven alkene reduction of different substrates using TsOYE and commercial ene-reductases ENE-103 and ENE-107.

Flavin-containing NAD^+^-linked SHs have long been known to have activity for reducing external flavin cofactors in solution,^42^ and this has been used to drive reactions for biotechnology where flavins can substitute for NAD(P)H,^43^ for example, the flavin-containing ene-reductases of the ‘old-yellow enzyme’ (OYE) type. More surprising was our finding that *E. coli* hydrogenase Hyd-1 also has a non-native activity for the reduction of the flavins FMN and FAD under H_2_ (Fig. 6(a)).^44^ The thermodynamic potential of the H^+^/H_2_ couple (−0.472 V, pH 8.0, 1 bar H_2_) compared to the flavin potential (around −0.230 V, pH 8.0, Fig. 6(a)) indicates that the reduction of flavins by H_2_ is thermodynamically favourable. The reaction likely occurs at the enzyme's surface where electrons from H_2_ oxidation are released *via* the outer iron–sulfur cluster of the electron relay chain (see Fig. 1(b)). The robustness of Hyd-1 allowed flavin reduction to be performed over a wide range of temperatures, 25–70 °C. To showcase the applicability of Hyd-1 in biotechnologically relevant flavin recycling, the Hyd-1-catalysed flavin reduction was coupled with the OYE-type ene-reductase from *Thermus scotoductus*, TsOYE, to achieve the enantioselective reduction of ketoisophorone to (*R*)-levodione (Fig. 6(b)). The Hyd-1 turnover frequency (TOF) reached only 20.4 min^−1^, well below the H_2_ oxidation rate measured electrochemically for this enzyme, likely due to the relatively low driving force for H_2_ oxidation provided by the flavin reduction half-reaction, as well as the non-native interaction of flavins with the FeS clusters at the enzyme surface. Engineering of a more targeted site for flavin reduction might offer improvements. Nevertheless, complete conversion of 2 mM of an alkene substrate of TsOYE was achieved after 15 hours at 0.5 mM FMN under mild conditions (room temperature and 1 bar H_2_) suggesting the promise of the H_2_-driven flavin recycling. Lower FMN concentration (0.1 mM) and higher substrate concentration (20–24.2 mM) made it possible to achieve a total turnover number (TTN) of up to 10200 for Hyd-1 and 97 FMN turnovers after 24 hours of reaction. This is similar to the FMN turnover number reported for formate-driven Rh-catalysed FMNH_2_ recycling.^45^ However, the background, non-enantioselective reduction of the substrate by [Cp*Rh(bpy)H]^+^ required careful catalyst balancing in that system. A fed-batch reaction confirmed that the system is stable for at least 134 h of reaction time, giving Hyd-1 TTN over 20 000 and FMN TN 240 – showing the advantage of the sturdy Hyd-1 biocatalyst over the *C. necator* SH for which TTN of 8400 was reported.

The Hyd-1 biocatalytic system was further demonstrated with two commercial ene-reductases, ENE-103 and ENE-107 from Johnson Matthey, which usually are run with GDH for NAD(P)H recycling. With the same protocols as previously optimised for TsOYE, two alkenes, dimethylitaconate (with ENE-103) and 4-phenyl-3-buten-2-one (with ENE-107), were reduced to dimethyl (*R*)-methyl succinate (>99% ee) and 4-phenyl-2-butanone, respectively (Fig. 6(b)). Control experiments confirmed the importance of each component of the system for the reduction of the corresponding alkene. These results emphasise the easy application of various ene-reductases with Hyd-1-catalysed flavin recycling, indicating that this simplified H_2_-driven system could be advantageous for applications requiring low waste, high catalyst stability, and good temperature tolerance.

This proof-of-concept study with Hyd-1 catalysed flavin recycling establishes the H_2_-driven reduction of FAD and FMN by hydrogenase as a viable alternative to recycling nicotinamide cofactors for enzymes that will tolerate alternative electron donors. The system's stability and temperature tolerance are promising for industrial biotechnology applications. The fact that FMN is significantly cheaper than NAD(P)H should make this system worth developing further for application with ene reductase catalysis.

In a follow-up study, we exploited the Hyd-1/H_2_ system to recycle FMN as a source of reducing equivalents for nitroreductases during the reduction of aromatic nitro compounds.^46^ Like ene reductases, the nitroreductases are flavoenzymes and are typically run with the standard glucose-driven NADH recycling system. The 6-electron reduction of a nitro group to the corresponding amine requires three equivalents of glucose, so the need for a cheaper, more atom-economical reductant is even more pertinent with these enzymes. Additional complications at this level of super-stoichiometric glucose are the formation of *N*-glucoside as a side product, and gluconolactone build-up, which requires constant pH monitoring and adjustment. We hypothesised that the nitro-reductases might also accept reducing equivalents from flavin in its reduced hydroquinone form, and that the drawbacks of glucose could be eliminated by using the hydrogenase/H_2_ flavin recycling system, which avoids pH changes, by-products, and side reactivity. The nitro reduction reaction proceeds *via* several partially reduced intermediates, and typically, nitroreductases reach hydroxylamines and often fail to fully convert substrate to the corresponding amine. This issue has been addressed by Dominguez and coworkers using V_2_O_5_ as a co-catalyst, which helps disproportionate the hydroxylamine intermediate and ultimately favours the formation of the amine product.^47^ When used with the V_2_O_5_ additive and the H_2_-driven flavin recycling system, the nitro reductase reactions achieve high conversion rates to the pure amine product.^46^

**Fig. 7:**
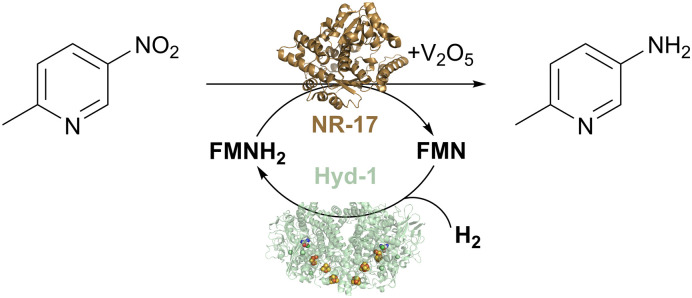
H_2_-driven biocatalytic reduction of the nitro compound to the corresponding amine using commercial nitroreductase NR-17, as described in ref. 46.

We tested the H_2_/Hyd-1/FMN system with a set of commercially available nitroreductases (from Johnson Matthey) to see if these enzymes could accept electrons from externally supplied FMNH_2_ instead of NAD(P)H. The model nitro aromatic substrate was used, 2-methyl-5-nitropyridine, which is known to reduce to the corresponding amine using nitroreductases with the glucose/GDH/NADP^+^ cofactor recycling system in the presence of the V_2_O_5_ cocatalyst (Fig. 7). Reactions were run at 10 mM substrate at pH 7.0, with 5% v/v DMSO as a co-solvent at 35 °C under a H_2_ atmosphere (1 bar). Analysis of reaction mixtures by gas chromatography showed that all tested nitroreductases converted the nitro substrate to the aniline product with the flavin hydroquinone as a reductant. One nitroreductase (NR-17) was chosen for further assessment of the catalyst system.^46^

Control experiments confirmed the importance of each component of the reaction mixture for selective conversion of substrate to corresponding amine. With an increased concentration of substrate (20 mM), a conversion of 96% was reached after 20 hours of reaction, and TTN for Hyd-1, in that case, was 26100 (taking into account the six-electron reduction of the substrate).^46^

To evaluate the waste reduction provided by the H_2_/Hyd-1/FMN system, we calculated an E-factor, which we compared to the published glucose/GDH/NADP^+^ system using a 20 mM concentration of nitroarene substrate. This indicated a more than 4-fold improvement by eliminating glucose from the process, and this could likely be improved further by reaction optimisation.^46^

This study also demonstrated that Hyd-1 effectively reduces the flavins FAD and FMN even in the presence of up to 50% co-solvent (DMSO or acetonitrile, MeCN), with higher specific activity observed for FMN compared with FAD. In DMSO, specific activity decreased somewhat up to 5% DMSO but remained stable as the co-solvent concentration was increased to 50%. Hyd-1 also showed stable FMN reduction activity between 5% and 50% MeCN. This tolerance to solvents could be crucial for enhancing system performance and expanding the substrate range to less water-soluble nitroaromatic compounds. In this study, we also reported a modest over-expression system for Hyd-1 in its native host *E. coli*.^46^ Together, these advances suggest that flavin recycling with Hyd-1/H_2_ is a promising system for the cleaner operation of flavoenzymes such as ene reductase and nitroreductases.

### Nitro reductions catalysed by hydrogenase on carbon

A strong demand for sustainable amine synthesis in pharmaceuticals and agrochemicals along with inspiration from the approach of ‘electrochemical hydrogenation’ in heterogeneous catalysis, underpinned a further application of Hyd-1 immobilised on carbon. Traditional nitro-group reductions to produce amines typically involve stoichiometric reductants or precious-metal hydrogenations, which lack functional group selectivity, although recent developments in organocatalysts and first-row transition metals have improved selectivity. Biocatalytic approaches using nitroreductase are still emerging, and substrate scope remains limited. Although Pd/C catalysts are widely used in nitro-group hydrogenations, they may cause unwanted side reactions. ‘Electrochemical hydrogenation’ involves coupling H_2_ oxidation to reduction at a different catalytic site, as seen with the PdCu alloy for nitrate reduction in Fig. 3(b). Indeed, An *et al.* have recently proposed a site-separated mechanism for nitro-reductions at Pd/C, whereby Pd is responsible for H_2_ oxidation, and the nitro-compound is reduced at the carbon support.^48^ The reduction of nitrobenzene, a model aromatic nitro compound, on a graphite electrode in aqueous medium at pH 6.0 begins at −0.113 V (eqn (6)).6
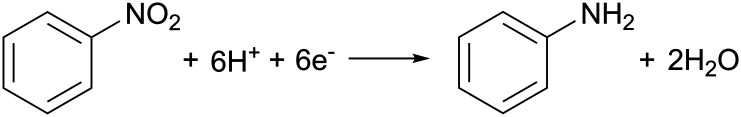


**Fig. 8:**
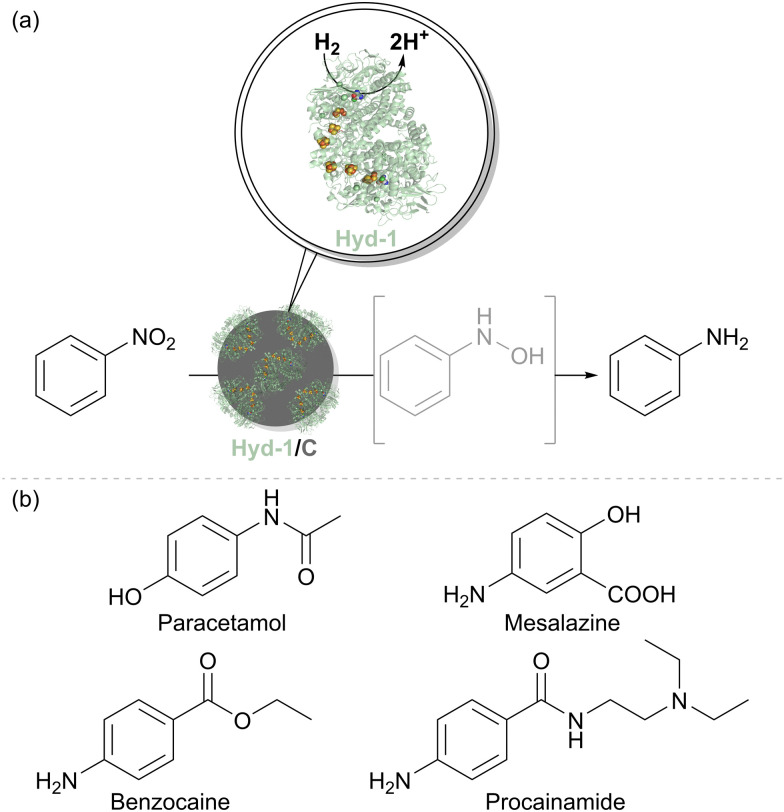
(a) H_2_-driven chemo-enzymatic reduction of nitro compound by Hyd-1/C catalyst. (b) Examples of amine-containing pharmaceuticals that can be produced by hydrogenation of corresponding nitro precursors using the Hyd-1/C catalyst.

At pH 6.0 and under 1 bar H_2_, the proton/dihydrogen couple potential, *E*′(2H^+^/H_2_), is −0.355 V. Since the onset potential for nitrobenzene reduction is more positive than *E*′(2H^+^/H_2_), reducing nitrobenzene with H_2_ is thermodynamically feasible. We, therefore, hypothesised that hydrogenase on a carbon support should be able to reduce nitroarene compounds, with reduction of the nitro group occurring at the carbon surface, similar to an electrochemical half-reaction, using electrons from H_2_ oxidation by hydrogenase (Fig. 8(a)).^49^*E. coli* Hyd-1 exhibits a small over-potential relative to *E*′(2H^+^/H_2_), with its H_2_ oxidation onset potential at about −0.296 V, but should still provide sufficient driving force for nitrobenzene reduction.

We therefore tested the feasibility of nitrobenzene hydrogenation using a Hyd-1/C catalyst, where the support is a carbon black material known as Black Pearls 2000 (Cabot). After 12 hours under H_2_ flow, a complete conversion of 10 mM nitrobenzene to aniline was observed with no side products. Control experiments confirmed that this reactivity is not exhibited by either Hyd-1 or carbon particles alone. It is likely that interfacing Hyd-1 with the carbon support aids the 6-electron reduction of the nitro compound by pooling electrons in the conductive support. Encouraged by these results, a broader range of aromatic nitro compounds was explored to assess the substrate scope, functional group tolerance, and chemoselectivity of the Hyd-1/C catalyst. Full hydrogenation of all 30 selected nitrobenzene derivatives to their corresponding amines was achieved by Hyd-1/C at 1 bar H_2_. In some cases, 10% v/v MeCN was required as a co-solvent to address solubility issues, and for others, reaction times or catalyst loading were increased to facilitate a complete conversion. These findings demonstrated the high tolerance of this biocatalyst system to various substituents on the aromatic ring. Of particular note, the Hyd-1/C catalyst hydrogenated nitro groups in several halogenated substrates without causing dehalogenation which often occurs at Pd/C,^50^ maintaining the halogen substituents (Cl, Br, I). The biocatalyst system also avoided side reductions in challenging substrates, such as benzylic alcohols and thiolate-containing compounds, and was selective for nitro hydrogenation over other unsaturated groups, such as ketones, aldehydes, or alkenes. Sterically hindered and bulky substrates were also effectively converted, although some required higher catalyst loadings or extended reaction times. Nitro compounds with two nitro groups were completely reduced to diamines. Additionally, we used the biocatalyst system to produce pharmaceutical precursors, including 4-aminophenol (for paracetamol), benzocaine, and mesalazine, an essential drug for treating inflammatory bowel disease (Fig. 8(b)).

After demonstrating Hyd-1/C as an effective catalyst for nitroarene reductions, the focus shifted to scaling up the reaction and isolating products. For most substrates, the corresponding amines were isolated by simple organic solvent extraction, yielding 78–96% product without further purification. Synthesis of the highest-yielding product, 1-naphthylamine, achieved 2.22 × 10^5^ turnovers of Hyd-1 during the 24-hour reaction.

To demonstrate the scalability of this biocatalytic system for producing a pharmaceutically relevant product, we selected the reduction of *N*-(2-(diethylamino)ethyl)-4-nitrobenzamide to procainamide, which is used to treat cardiac arrhythmia. The precursor was hydrogenated using Hyd-1/C, yielding 1.10 g of procainamide with 96% purity and 90% yield. This result demonstrated the system's scalability and potential for application in the production of fine chemicals and their precursors.

To explore the mechanism of nitro-group hydrogenation with Hyd-1/C and H_2_, we examined the reduction of nitrobenzene as a model substrate, taking time points during the reaction. After 30 minutes, *N*-phenylhydroxylamine was detected as an intermediate, with complete conversion to aniline occurring by 12 hours. Cyclic voltammetry confirmed the formation of *N*-phenylhydroxylamine, indicating a four-electron reduction pathway from nitrobenzene to *N*-phenylhydroxylamine, followed by further two-electron reduction to aniline. Catalyst recycling experiments showed complete conversion over five cycles of the catalyst re-use, with a total turnover number (TTN) of 1.16 × 10^6^. Despite some increase in the *N*-phenylhydroxylamine intermediate over thirteen cycles, the starting material was fully consumed in each cycle.

We used cyclic voltammetry to examine all of the nitroarene substrates tested in the hydrogenation reactions and found that all had reduction onset potentials positive relative to both *E*′(2H^+^/H_2_) and the H_2_ oxidation onset for Hyd-1. However, the aliphatic nitro compound, 1-nitrohexane, with a more negative onset potential of −0.313 V, failed to reduce using the Hyd-1/C catalyst, suggesting a potential limit for hydrogenation of substrates by Hyd-1/C.

To address this, we substituted in *E. coli* hydrogenase 2, Hyd-2, which operates reversibly at *E*′(2H^+^/H_2_) (*i.e.* without the kinetic limitation that gives an overpotential requirement to Hyd-1). As expected, over 48 hours, Hyd-2/C converted 1-nitrohexane to 1-aminohexane, indicating that the ‘hydrogenase on carbon’ catalyst concept can be extended to more challenging aliphatic nitro compounds.

The fact that a total turnover number of over 1 million is achieved for Hyd-1/C without significant optimisation, suggests that this system should be adaptable for industrial application. The enzyme immobilised on a carbon support can be handled similarly to Pd/C and hence should ‘slot in’ to existing reactors. The ability of hydrogenase on carbon to catalyse the selective hydrogenation of nitro compounds allows for Pd-free ‘electrochemical hydrogenation’ of nitro compounds – a new paradigm for industrial biotechnology. It will be interesting to see what further opportunities emerge for reactions of this type using Hyd-1, which is sufficiently solvent-tolerant, temperature-stable and robust.

## Scalability and future scope

Scale-up of NiFe hydrogenase production remains a significant challenge for the application of these enzymes in biotechnology, although new approaches are emerging (*vide infra*). Heterologous expression of NiFe hydrogenases is hindered by the number of accessory genes required for successful assembly of the active site and maturation of the protein, and these may be specific to a particular hydrogenase. For example, *E. coli* expression of the regulatory hydrogenase from *Cupriavidus necator* (formerly known as *Ralstonia eutropha*) was achieved with co-expression of six maturation proteins, HypABFCDE, alongside the subunits of the hydrogenase dimer, HoxBC.^51^ The number of accessory proteins also hinders over-expression within the native organism, although we did achieve ca 10-fold over-expression of Hyd-1 in its native host, *E. coli*.^46^ Substantial efforts in synthetic biology and bioprocess engineering are likely to be necessary to achieve high-level bioreactor expression of active NiFe hydrogenases.^52^

For the FeFe hydrogenases, an interesting opportunity exists because it has been possible to express apo hydrogenase (with no diiron active site) at higher levels in *E. coli*, and then incorporate a chemically-synthesised, small molecule diiron cluster to generate active FeFe hydrogenase. For the FeFe hydrogenases, maturases have more cross-compatibility, and it has also been possible to establish a strain of *E. coli* with the maturase from *Clostridium acetobutylicum* to allow expression of intact, holo FeFe hydrogenase from other organisms.^53^ It remains to be seen whether the inorganic synthesis of the diiron active site precursor is itself sufficiently scalable to enable the former approach to be used in large-scale biotechnology, or whether enabling *E. coli* to biosynthesise the cofactor *via* the introduction of the maturases is preferable. The extreme air-sensitivity of FeFe hydrogenases also hinders their preparation, although inhibition by sulfide coordination at the active site has been shown to offer temporary protection during aerobic handling prior to reactivation by removal of sulfide.^54,55^

The FeFe hydrogenase, HydA5 from *Clostridium* (*C.*) *beijerinckii*, shows substantially more stability towards O_2_ than other known FeFe hydrogenases, attributed to a cysteine residue which swings in to confer additional sulfur coordination at the active site to block O_2_ reaction, facilitating aerobic purification of the enzyme.^56^ Although this process also limits the enzyme activity to a minimal potential window close to the onset of H_2_ oxidation, *C. beijerinckii* HydA5 has been shown by Morra, Cleary and coworkers to be viable for H_2_-driven flavin reduction to support ene-reductase catalysis by an old yellow enzyme type ene-reductase,^57^ similar to the activity which was discussed earlier for NiFe hydrogenase. In combination with an NAD^+^ reductase moiety on a carbon support, *C. beijerinckii* HydA5 has also been used for H_2_-driven NADH recycling to support an NADH-dependent alcohol dehydrogenase for the production of the pharmaceutical precursor quinuclidinol with a total turnover number of 135 300 over an 18-hour reaction, and near-complete conversion of almost 50 mM substrate.^57^ Small-scale heterologous production of this enzyme in *E. coli* in a bioreactor looks promising for applications in biotechnology, in this case *via* heterologous expression in a strain of *E. coli* that has been engineered to incorporate a set of maturases.^57^ It therefore remains to be seen whether FeFe hydrogenases will catch up with the NiFe enzymes in terms of applicability for biotechnology. The high activities of hydrogenases (often at least >1000 s^−1^ for H_2_ oxidation) slightly mitigate the challenges in enzyme expression because a little of the enzyme goes a long way.

## Conclusions

Challenges of enzyme supply aside, the catalytic systems involving hydrogenase in solution or on a carbon support are inherently scalable because they can slot directly into reactors designed for homogeneous or heterogeneous hydrogenations which are widely used in chemical manufacturing. The high affinity of hydrogenases for H_2_ means that reactions can be performed at atmospheric or mild pressures of H_2_, avoiding complex high-pressure reactor infrastructure which is required for many metal-catalysed hydrogenations, hence offering additional safety benefits. The ability of hydrogenases to tolerate contaminants in the H_2_ stream, such as CO or H_2_S offers possibilities of running reactions on lower-grade H_2_ or waste streams from other industrial processes. Future applications are likely to exploit further native electron transfer pathways of hydrogenases, for example, the soluble hydrogenases for NADPH or deazaflavin (F_420_) cofactor recycling, as well as non-native activities beyond the flavin and nitro reductions. Further reactivities are likely to emerge, paralleling the conceptual developments in ‘electrochemical hydrogenations’ in the field of heterogeneous catalysis. We can expect hydrogenases to play an increasing role in cleaner, more sustainable biotechnology.

## Data availability

No primary research results, software or code have been included and no new data were generated or analysed as part of this article.

## Conflicts of interest

K. A. V. is a co-founder and director of the company HydRegen which holds licences for several of the biocatalyst systems described in this Feature Article. Patents have been filed on several of the technologies described in this Feature and the authors may benefit from future royalties. The authors declare no further conflicts of interest and there are no unpublished results disclosed in this Article.
